# A novel species of torque teno mini virus (TTMV) in gingival tissue from chronic periodontitis patients

**DOI:** 10.1038/srep26739

**Published:** 2016-05-25

**Authors:** Yu Zhang, Fei Li, Tong-Ling Shan, Xutao Deng, Eric Delwart, Xi-Ping Feng

**Affiliations:** 1 Department of Preventive Dentistry, Ninth People’s Hospital, School of Medicine, Shanghai Jiao Tong University, Shanghai Key Laboratory of Stomatology, Shanghai, China; 2 Department of Swine Infectious Disease, Shanghai Veterinary Research Institute, Chinese Academy of Agricultural Sciences, Shanghai, China; 3 Blood Systems Research Institute, San Francisco, California, USA; 4 Department of Laboratory Medicine, University of California at San Francisco, San Francisco, California, USA

## Abstract

A new species of torque teno mini virus, named TTMV-222, was detected in gingival tissue from periodontitis patients using a viral metagenomics method. The 2803-nucleotide genome of TTMV-222 is closely related to TTMV1-CBD279, with 62.6% overall nucleotide similarity. Genetic analyses of the new virus genome revealed a classic genomic organization but a weak identity with known sequences. The prevalence of TTMV-222 in the periodontitis group (n = 150) was significantly higher than that in the healthy group (n = 150) (*p* = 0.032), suggesting that the new virus may be associated with inflammation in chronic periodontitis patients. However, this finding requires further investigation.

Periodontitis is a common, multifactorial, chronic oral disease that can lead to the early loss of teeth. While the incidence of periodontal disease is high, the etiology remains unclear. Multiple infectious agents are believed to play a role in the pathogenesis of this disease. Bacteria, such as *Porphyromonas gingivalis, Tannerella forsythia*, *Aggregatibacter actinomycetemcomitans*, and *Treponema denticola,* are thought to be major causes of periodontal disease^1^,^2^,^3^,^4^,^5^, but the bacterial theory cannot fully explain all of the clinical symptoms associated with periodontal disease^6^,^7^,^8^. Despite the presence of abundant periodontopathic bacteria in the oral cavity, periodontitis is a site-specific disease. The disease course includes remission periods that can be interrupted by episodes of clinical relapses^9^. The latent biological basis of this phenomenon is not clearly understood. The possible role of viruses in periodontal disease episodes has begun to attract more attention^10^. Viruses have been implicated in the pathogenesis of periodontal diseases since the mid-1990s^11^,^12^,^13^,^14^,^15^. Previous data have shown that individual periodontal lesions harbor millions of genomic copies of herpes viruses, papilloma viruses, human T-lymphotropic virus-1^11^, hepatitis B virus^12^, hepatitis C virus^13^, Human Immunodeficiency Virus (HIV) and torque teno virus (TTV)^14^. Chikungunya virus was also reported to be associated with Indian gingivitis patients^15^. Recent studies have discussed the possible role of bacteriophages in periodontal disease patients, suggesting that oral phages may be more highly expressed in periodontally healthy subjects and that periodontitis may be beneficial for the expression of some lytic phages^1^,^16^. All of these studies have provided new insight into the possible cause of this disease.

Viral metagenomics is able to characterize viral communities and to detect previously uncharacterized viruses based on sequence similarities to previously sequenced viral genomes^17^,^18^,^19^,^20^. This strategy has been used to discover a wide diversity of viruses in both marine and freshwater environments and in human and animal samples^21^,^22^,^23^,^24^,^25^,^26^,^27^,^28^,^29^. In the present study, we characterized a novel human anellovirus from chronic periodontitis patients in China using viral metagenomics technology. Epidemiological strategies were adopted to explore the correlation between the newly identified virus and chronic periodontitis.

## Results

### Discovery of a novel TTMV

A total of 48 chronic periodontitis subjects (24 men and 24 women) with a mean age of 42.2 years of age were included in this study. All of them received a periodontal examination at the Ninth People’s Hospital, School of Medicine, Shanghai Jiao Tong University in China. Each diagnosis was based on clinical and radiographic findings according to the inclusion standards described in the Methods. The mean pocket depth (PD), clinical attachment loss (CAL), gingival index (GI), and plaque index (PLI) of all of the participants were 8.55 mm, 4.52 mm, 2.27, and 2.58, respectively. Samples were taken from each of the patients. The viral particles and their nucleic acids in these samples were enriched by filtration and nuclease treatment before the nucleic acids were extracted for use in random RT-PCR. The samples were then sequenced using the MiSeq platform. Based on the best BLAST score (*E* < 0.001), a novel viral sequence (222 bp), identified as an anellovirus, was further analyzed. This sequence had a 39% amino acid homology with a known human anellovirus.

### Full-length nucleotide sequences of the newly identified virus

**Figure 1:**
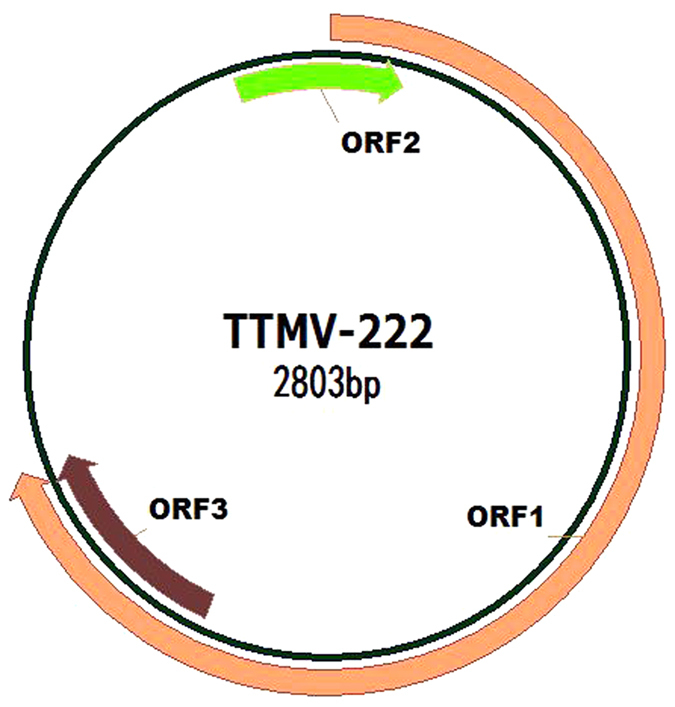
The genome organization of TTMV-222. Annotations and illustritions were made using Vector NTI 10.

Based on the novel viral 222-bp sequencent, primers (AL1 5′-A*C*TCGTGTGTCTCCTCCTCG-3′, AR1 5′-C*G*GAGACGGCATCACATCAG-3′, AR2 5′-TGATACCCGGCTGATCTTGG-3′) were designed for amplification by inverse PCR and Sanger sequencing. The complete genome of the new isolate was 2803 bp in length and contained three open reading frames (ORFs) (Fig. 1). ORF1 is 1941 bp (nt316–2256) in length and encodes a 646-amino acid protein. ORF2 is 267 bp (nt170–436) in length and encodes an 88-amino acid protein. This ORF partially overlaps with ORF1 (nt316–436). ORF3 is 273 bp (nt1979–2251) in length, encodes a 90-amino acid protein, and also overlaps with ORF1 (nt1979–2251). These characteristics are consistent with the composition of the human TTMV strains that have been reported^30^,^31^. The viral genome of the new isolate was deposited into GenBank (accession number: KU041847) and was named TTMV-222.

### Sequence analysis of TTMV-222

**Figure 2:**
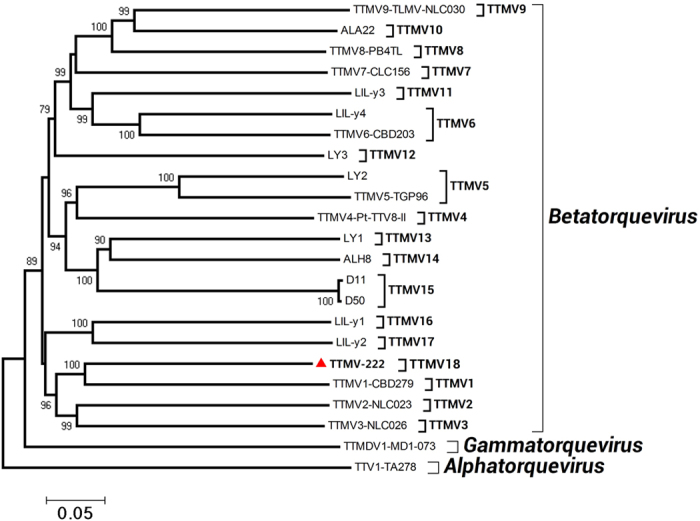
ORF1 nucleotide phylogenetic tree constructed using the neighbor-joining method with nucleotide p distances and 1,000 bootstrap replicates in the Molecular Evolutionary Genetics Analysis program (MEGA, version 4.0, USA). Bootstrap values are indicated at each branching point. The solid triangles indicate the noval TTMV species.

A phylogenetic tree (Fig. 2) was constructed with ORF1 from TTMV-222 and TTMV reference species^31^. TTMV-222 is related to TTMV1-CBD279, TTMV2-NLC023, and TTMV3-NLC026, all of which belong to the family torque teno mini virus (TTMV), genus *Betatorquevirus* (Fig. 2). Homology analysis revealed that TTMV-222 has 62.6% and 55.8% nucleotide homologies with the TTMV1-CBD279 (GenBank accession No. AB026931) and TTMV2-NLC023 strains (GenBank accession No. AB038629), respectively (Table 1). In ORF1, TTMV-222 shares the highest nucleotide homology (60.4%) with the TTMV1-CBD279 strain; TTMV-222 ORF1 also has 47.3% amino acid homology with this strain (Table 1). The nucleotide and amino acid homologies of TTMV-222 with reference strains of TTMV for ORF1, ORF2, ORF3, and the complete genome are listed in Table 1. The nucleotide homologies between different TTMV species for ORF1 are shown in Table S1 in the supplemental material.

### Investigation of TTMV-222 prevalence

A case control study was conducted to investigate the association between the prevalence of the newly identified virus and chronic periodontitis. We enrolled 150 chronic periodontitis patients (mean age 45) and 150 periodontal healthy participants (mean age 40) in a case control study. Each diagnosis was based on clinical and radiographic findings according to the inclusion standards described in the Methods. Of the 300 total subjects, no differences in the age or gender distribution were noted between the periodontitis group and the control group (*p* = 0.647 and 0.729, respectively). Mean PD values, mean CAL, mean PLI, mean GI and the percentage of subjects with bleeding on probing (BOP) were significantly higher in participants with periodontitis than in the healthy group. These data are shown in Tables S2 and S3 in the supplemental material.

Based on the amplified full-length sequence, primers (DL1 5′-T^*^G*AGTGAAACCACCGAAGTC-3′, DR1 5′-C*G*TTACTTGTTGTCCACCAG-3′, DL2 5′-ACCACGGATTATTCTGCGGC-3′, and DR2 5′-AAAAGACCATGCTCCCCCTC-3′) were designed to screen the samples from the chronic periodontitis patients and periodontally healthy subjects for the novel TTMV. The TTMV-222 virus was detected in 6.00% (9/150) and 1.33% (2/150) of chronic periodontitis patients and periodontally healthy subjects, respectively. The difference in the TTMV-222 prevalence between the two groups was statistically significant (*p* = 0.032), suggesting that detection of TTMV-222 may be associated with chronic periodontitis, although this finding requires further investigation. Relationships between other factors and detection of TTMV-222 were also detected (Tables 2 and 3). No correlations with age or gender distribution (*p* = 0.496 and 0.290, respectively) were noted.

## Discussion

Chronic periodontitis, which can damage the periodontal tissue and result in the early loss of human teeth, is among the most prevalent infectious diseases worldwide^32^. The causative agents of periodontal disease are generally thought to be periopathogenic bacteria, although the pure bacterial pathogenicity theory cannot cover all of the manifestations of periodontal diseases. Increasing evidence also support a role for pathogens other than bacteria in the pathogenesis of periodontal disease^7^,^9^,^10^. Recently, research into viruses as periodontal pathogenic factors has garnered much attention^1^,^16^,^33^,^34^,^35^, especially for herpesviruses. Published evidence has strongly suggested that Epstein-Barr virus (EBV) and human cytomegalovirus (HCMV) are implicated in the pathogenesis of periodontal disease^2^,^5^,^7^,^9^,^10^. Furthermore, bacteriophages may also play a role in periodontitis, and periodontitis may be beneficial for the expression of some lytic phages^16^. Nevertheless, the composition of the viral community in the periodontal environment and its association with periodontal diseases is still poorly understood. To investigate the makeup of this community, we applied an unbiased viral metagenomics method to characterize the periodontal virome^17^,^19^,^20^.

Human anelloviruses were first discovered in 1997 and have been classified into three genera in the family *Anelloviridae*: TTV, genus *Alphatorquevirus*; TTMV, genus *Betatorquevirus*; and torque teno midi virus (TTMDV), genus *Gammatorquevirus*^36^,^37^. Their genomes consist of small, single-stranded circular DNA, and their capsids have no lipid envelopes. The genomes of anelloviruses range in size from 2.1 to 3.9 kb in length and contain three or four overlapping ORFs and a short, GC-rich un-translated region^38^. Recent studies have indicated the existence of multiple anellovirus co-infections and interactions between TTVs and the host immune system^39^,^40^.

In the present study, a new human anellovirus, TTMV-222, was detected by viral metagenomics in gingival tissue from periodontitis patients. Phylogenetic analysis showed that this newly characterized genome belonged to the TTMV genus. Our first phylogenetic tree analysis, which was based on the known TTMV genomic sequences, showed a large cluster of strains that diverged considerably from each other in ORF1 by over 42% at the nucleotide level and over 67% at the amino acid level^41^,^42^. Three visible clusters were evident in the phylogenetic tree: TTV, TTMDV, and TTMV. TTMV-222 was genetically closest to the TTMV1-CBD279 strain (GenBank accession No. AB026931) and the TTMV2-NLC023 strain (GenBank accession No. AB038629). At the nucleotide level, the full-length sequence of TTMV-222 shared 37.4% and 44.2% sequence heterogeneity while the TTMV-222 ORF1 shared 39.6% and 42.3% sequence heterogeneity with the TTMV1-CBD279 and TTMV2-NLC023 strains, respectively. At the amino acid level, TTMV-222 ORF1 shared 52.7% and 58.7% heterogeneity with the TTMV1-CBD279 and TTMV2-NLC023 strains, respectively (Table 1). The A% nucleotide similarities between TTMV species (TTMV1-9,TTMV-222) for ORF1 are also shown in Table S1. We propose that TTMV-222 may be a new species of torque teno mini virus.

In addition, we investigated the prevalence of TTMV-222 in chronic periodontitis patients and periodontally healthy participants. The TTMV-222 prevalence in the chronic periodontitis group was significantly higher than in the healthy group (*p* = 0.032), suggesting that TTMV-222 may be more likely to be found in chronic periodontitis patients than in healthy individuals. However, the detection rate was too low to provide a robust connection between chronic periodontitis and TTMV. Several reasons might account for this finding. First and foremost, all samples from chronic periodontitis patients were collected after initial therapy, including supergingival and subgingival scaling. To the best of our knowledge, these treatments would certainly relieve the severity of periodontitis and would reduce the number of viruses^43^,^44^. Secondly, the reported prevalence rates of TTMV are significantly different among geographical regions. Data from Brazil^45^, Russia^46^, Japan^47^ and Pakistan^48^ have described prevalence rates varying from 5% to 90%, suggesting that the TTMV prevalence might be related to ethnicity. The epidemic situation of other races requires further investigation. The potential robust association between TTMV and chronic periodontitis requires further investigation.

In summary, this report describes a novel TTMV species from periodontal pockets, which we named TTMV-222. The present study demonstrated the effectiveness of viral metagenomics for the discovery of emerging viruses in the periodontal environment and provided new evidence to implicate viruses in periodontal diseases.

## Methods

### Sample collection

Patients attending the dental clinics at the Ninth People’s Hospital, School of Medicine, Shanghai Jiao Tong University, China between June 2014 and June 2015 were invited to participate in this study. All participants underwent a comprehensive clinical periodontal examination including X-rays. All participants were systemically healthy, with a minimum of 20 teeth present (excluding third molars). A total of 48 infected gingival epithelium samples of the periodontal pocket from 48 severe periodontitis patients (24 men and 24 women), aged 18–65 years, were collected for high-throughput sequencing. Inclusion criteria for this study were: mean CAL ≥3 mm, mean PD ≥6 mm, BOP, and alveolar bone resorption detected using pantomography^49^. Exclusion criteria were: diabetes, heart disease, human immunodeficiency virus infection, pregnancy, heavy cigarette smoking (>15 cigarettes/day), previous periodontal treatment (within the previous 6 months), and previous antibiotic intake (within the previous 6 months). Biopsy specimens were periodontal pockets or the gingival epithelium and connective tissue facing the sulcus, which were obtained during the surgical phase aimed at pocket elimination. The samples were rinsed five times with phosphate-buffered saline (PBS) to wash away blood, saliva, and plaque and were then immediately placed in an Eppendorf tube containing 500 μl PBS and transported to the laboratory and stored at −80 °C for analysis. All participants signed a written informed consent form before their inclusion in the study. The Ethics Committee of the Ninth People’s Hospital, School of Medicine, Shanghai Jiao Tong University approved this study. The study was performed in accordance with the principles of the Declaration of Helsinki.

Subjects aged 18–65 years with severe periodontitis (150 total) and 150 periodontal healthy volunteers also aged 18–65 years were recruited for the epidemiological investigation. In the periodontitis group, the inclusion and exclusion standards were the same as those described above, and biopsy specimens were obtained in the same manner. In the control group, participants showed no evident clinical signs of gingival inflammation and had no detectable PD ≤4 mm, no CAL, and no BOP and were considered periodontally healthy. Tissue samples were the gingival epithelium and connective tissue facing the sulcus from periodontally healthy sites. Biopsies were obtained from the sulcular region during a tooth extraction procedure. Some of the subjects in this group exhibited isolated sites with BOP, but for the study purposes, only sites with no BOP were sampled. A full-mouth clinical examination was conducted in each subject. PD (in mm), CAL (in mm), BOP, the GI, and the PLI of each subject were recorded.

### Identification of a novel anellovirus by viral metagenomics

The samples were vortexed with small magnetic beads for 5 min and freeze-thawed three times. After centrifugation (10 min at 13,000 × g), 300 μl of supernatant was collected and filtered through a 0.45-μm filter to remove eukaryotic and bacterial cell-sized particles. To digest unprotected nucleic acids (not in viral capsids), a mixture of DNases (Turbo DNase from Ambion, MA, USA, Baseline-ZERO from Epicentre, IL, USA, and benzonase from Novagen, Darmstadt, Germany) and RNase (Thermo Fisher Scientific, MA, USA) was added to the filtrates, which were enriched in viral particles, and the samples were incubated at 37 °C for 90 min^50^. Viral nucleic acids were then extracted using a QIAamp viral RNA extraction kit (QIAGEN, Dusseldorf, Germany) according to the manufacturer’s instructions, protected from degradation by the addition of an RNase inhibitor (Thermo Fisher Scientific, MA, USA), and stored at −80 °C for future processing. Viral nucleic acid libraries containing both DNA and RNA viral sequences were constructed by random RT-PCR amplification based on the Nextera XT DNA Sample Preparation Kit (Illumina, CA, USA). The libraries were sequenced using the MiSeq platform. The sequencing reads were assigned to 48 data bin-based barcodes. Trimmed sequences from each group were assembled into contigs using the method described by Eric^51^ in 2011, with a criterion at least 95% identity over 35-bp to merge two fragments. The assembled contigs and singlet sequences were then compared to GenBank using BLASTx. Sequences with E values of ≤10^−5^ in a BLASTx search were classified as likely originating from a eukaryotic virus, bacterium, phage, eukaryote, other, or unknown based on the taxonomic origin of the sequence with the best E value.

### Amplifications of the full-length newly discovered human anellovirus

The full-length anellovirus was amplified by inverted nested PCR based on the sequence obtained from the MiSeq analysis. An overlapping PCR fragment including the remainder of the circular genome (containing the GC-rich region) was obtained and then sequenced. Amplification was performed using Takara LA Taq polymerase and GC buffer I (LA PCR Kit Ver.2.1, TaKaRa, Dalian, China) and was performed as follows: 94 °C for 3 min, followed by five cycles of 1 min at 94 °C, 1 min at 60 °C, and 3.5 min at 72 °C, and then 30 cycles of denaturation at 94 °C for 30 s, annealing at 55 °C for 30 s, and extension at 72 °C for 3.5 min, with an additional 1 s in the extension stage every cycle and a final extension step of 10 min at 72 °C. The second-round cycling conditions were the same as above. The PCR products were excised from 1% agarose gels containing ethidium bromide (0.5 g/ml) and were purified using an AxyPrep DNA Gel Extraction Kit (Axygene, Silicon Valley, USA) according to the manufacturer’s instructions.

### Phylogenetic analysis and sequence similarity analysis

The genome sequence of the newly discovered human anellovirus isolated in this study was aligned using ClustalW with the default settings, and the aligned sequences were trimmed to match the human anellovirus sequences obtained from the human anelloviruses detected using ClustalW. Sequence analyses were performed with MegAlign software (DNAStar Inc., Madison, WI, USA). A phylogenetic tree was constructed using the neighbor-joining method with nucleotide p distances and 1,000 bootstrap replicates in the Molecular Evolutionary Genetics Analysis program (MEGA, version 4.0, USA) and by inputting the alignment of the ORF1 genome sequences^31^. Bootstrap values are indicated at each branching point. A % similarity analysis was also conducted using the MEGA program.

### Prevalence investigation

DNA from 300 clinical samples was extracted using a QIAamp DNA Mini kit (QIAGEN, Dusseldorf, Germany) according to the manufacturer’s instructions. Nested PCR assays were performed with a final volume of 50 μl of reaction mixture consisting of 2 μl of extracted DNA from the clinical sample, 25 μl of PrimerSTAR Max Premix, and 10 pmol of primers. The nested PCR parameters were 3 min at 94 °C, followed by 30 s at 94 °C, 30 s at 55 °C, and 50 s at 72 °C for 35 cycles, with a final extension of 5 min at 72 °C. The second-round cycling conditions were the same as described above. Amplification products were resolved by electrophoresis on 1% agarose gels and then sequenced. The detection frequency (%) of the new anellovirus-positive subjects was calculated.

### Statistical analyses

All statistical analyses were conducted using SPSS software (ver. 20.0; IBM Corporation, Armonk, NY, USA) with the significance level set to *p* < 0.05. Descriptive statistics (means, standard deviations, and percentages) were calculated for subjects’ socio-demographic characteristics (gender and age). Chi-squared and Fisher exact tests were performed to examine differences in the prevalence of the newly discovered virus and in one of the clinical indexes (BOP) between the case group and the control group. Student’s *t*-tests were used to examine differences in other clinical indexes (PD, GI, PLI, and CAL).

## Additional Information

**How to cite this article**: Zhang, Y. *et al.* A novel species of torque teno mini virus (TTMV) in gingival tissue from chronic periodontitis patients. *Sci. Rep.* **6**, 26739; doi: 10.1038/srep26739 (2016).

## Supplementary Material

Supplementary Information

## Figures and Tables

**Table 1:** Sequence similarity of TTMV-222 with other anellovirus strains.

TTMV strain (Genbank accession NO.)	Complete genome, %	Tree Shrew TTMV-222			(KU041847)		
		Nucleotides, %			Amino acids, %		
		ORF1	ORF2	ORF3	ORF1	ORF2	ORF3
TTMV1-CBD279 (AB026931)	62.6	60.4	60.3	56.2	47.3	43.7	44.4
TTMV2-NLC023 (AB038629)	55.8	57.7	62.7	57.1	41.3	46.5	35.6
TTMV3-NLC026 (AB038630)	56.8	57.5	56.2	61.6	38.3	48.8	40.4
TTMV-LIL-y1 (EF538880)	60.2	59.1	52.9	46.4	34.8	49.4	28.9
TTMV_LY1 (JX134044)	54.3	51.1	49.8	52.8	22.8	28.4	32.2
TTMV_LY2 (JX134045)	54.9	51.5	50.4	45.3	31.9	33.3	29.2
TLMV9-NLC030 (AB038631)	52.6	49.1	49.2	59.6	31.2	32.9	38.9
TLMV6-CBD203 (AB026929)	55.8	51.7	51.6	50.6	32.2	36.6	31.1

**Table 2:** Relationships between age and TTMV-222.

Group	TTMV-222(+) (mean ± SD)	TTMV-222(−) (mean ± SD)	*p*
Age	45.00 ± 15.80	42.52 ± 11.69	0.496

Obtained by Student’s *t*-test (two groups). ^a^*p* < 0.05.

SD: standard deviation.

**Table 3:** Relationships between gender, priodontitis and TTMV-222.

Group	*n*	% with TTMV-222(+)	*p*
Gender			0.290
Male	7	4.9	
Female	4	2.6	
Priodontitis			0.032
Positive	9	6.0	
Negative	2	1.3	

Obtained by Chi-squared test. ^a^*p* < 0.05.

## References

[b1] Ly M. *et al.* Altered oral viral ecology in association with periodontal disease. MBio 5, e01133–01114, 10.1128/mBio.01133-14 (2014). 24846382 PMC4030452

[b2] Abusleme L. *et al.* The subgingival microbiome in health and periodontitis and its relationship with community biomass and inflammation. ISME J 7, 1016–1025, 10.1038/ismej.2012.174 (2013). 23303375 PMC3635234

[b3] Paster B. J. & Dewhirst F. E. Molecular microbial diagnosis. Periodontol 2000 51, 38–44, 10.1111/j.1600-0757.2009.00316.x (2009). 19878468 PMC3070264

[b4] Socransky S. S. & Haffajee A. D. Periodontal microbial ecology. Periodontol 2000 38, 135–187, 10.1111/j.1600-0757.2005.00107.x (2005). 15853940

[b5] Feng Z. & Weinberg A. Role of bacteria in health and disease of periodontal tissues. Periodontol 2000 40, 50–76, 10.1111/j.1600-0757.2005.00148.x (2006). 16398685

[b6] Umeda M., Contreras A., Chen C., Bakker I. & Slots J. The utility of whole saliva to detect the oral presence of periodontopathic bacteria. J Periodontol 69, 828–833, 10.1902/jop.1998.69.7.828 (1998). 9706862

[b7] Goodson J. M., Tanner A. C., Haffajee A. D., Sornberger G. C. & Socransky S. S. Patterns of progression and regression of advanced destructive periodontal disease. J Clin Periodontol 9, 472–481 (1982). 6960023 10.1111/j.1600-051x.1982.tb02108.x

[b8] Hugoson A., Sjodin B. & Norderyd O. Trends over 30 years, 1973–2003, in the prevalence and severity of periodontal disease. J Clin Periodontol 35, 405–414, 10.1111/j.1600-051X.2008.01225.x (2008). 18433384

[b9] Ambili R. *et al.* Viruses: are they really culprits for periodontal disease? A critical review. J Investig Clin Dent 5, 179–187, 10.1111/jicd.12029 (2014). 23447363

[b10] Das S., Krithiga G. S. & Gopalakrishnan S. Detection of human herpes viruses in patients with chronic and aggressive periodontitis and relationship between viruses and clinical parameters. J Oral Maxillofac Pathol 16, 203–209, 10.4103/0973-029X.98502 (2012). 22923891 PMC3424935

[b11] Soto-Ramirez L. E. *et al.* Human T-lymphotropic virus type I (HTLV-I)-specific antibodies and cell-free RNA in crevicular fluid-rich saliva from patients with tropical spastic paraparesis/HTLV-I-associated myelopathy. Viral Immunol 8, 141–150 (1995). 8833267 10.1089/vim.1995.8.141

[b12] Bass B. D., Andors L., Pierri L. K. & Pollock J. J. Quantitation of hepatitis B viral markers in a dental school population. J Am Dent Assoc 104, 629–632 (1982). 6951863 10.14219/jada.archive.1982.0281

[b13] Maticic M. *et al.* Detection of hepatitis C virus RNA from gingival crevicular fluid and its relation to virus presence in saliva. J Periodontol 72, 11–16, 10.1902/jop.2001.72.1.11 (2001). 11210067

[b14] Rotundo R. *et al.* TT virus infection of periodontal tissues: a controlled clinical and laboratory pilot study. J Periodontol 75, 1216–1220, 10.1902/jop.2004.75.9.1216 (2004). 15515336

[b15] Katti R., Shahapur P. R. & Udapudi K. L. Impact of Chikungunya virus infection on oral health status: an observational study. Indian J Dent Res 22, 613, 10.4103/0970-9290.90325 (2011). 22124070

[b16] Santiago-Rodriguez T. M. *et al.* Transcriptome analysis of bacteriophage communities in periodontal health and disease. BMC Genomics 16, 549, 10.1186/s12864-015-1781-0 (2015). 26215258 PMC4515923

[b17] Alavandi S. V. & Poornima M. Viral metagenomics: a tool for virus discovery and diversity in aquaculture. Indian J Virol 23, 88–98, 10.1007/s13337-012-0075-2 (2012). 23997432 PMC3550753

[b18] Adams I. P. *et al.* Next-generation sequencing and metagenomic analysis: a universal diagnostic tool in plant virology. Mol Plant Pathol 10, 537–545, 10.1111/j.1364-3703.2009.00545.x (2009). 19523106 PMC6640393

[b19] Djikeng A. *et al.* Viral genome sequencing by random priming methods. BMC Genomics 9, 5, 10.1186/1471-2164-9-5 (2008). 18179705 PMC2254600

[b20] Edwards R. A. & Rohwer F. Viral metagenomics. Nat Rev Microbiol 3, 504–510, 10.1038/nrmicro1163 (2005). 15886693

[b21] Zhang T. *et al.* RNA viral community in human feces: prevalence of plant pathogenic viruses. PLoS Biol 4, e3, 10.1371/journal.pbio.0040003 (2006). 16336043 PMC1310650

[b22] Anderson R. E., Sogin M. L. & Baross J. A. Evolutionary strategies of viruses, bacteria and archaea in hydrothermal vent ecosystems revealed through metagenomics. PLoS One 9, e109696, 10.1371/journal.pone.0109696 (2014). 25279954 PMC4184897

[b23] M. D., Nakamura S., Hagiwara K. & Nakaya T. Viral detection by high-throughput sequencing. Methods Mol Biol 1236, 125–134, 10.1007/978-1-4939-1743-3_11 (2015). 25287501

[b24] Willner D. *et al.* Metagenomic analysis of respiratory tract DNA viral communities in cystic fibrosis and non-cystic fibrosis individuals. PLoS One 4, e7370, 10.1371/journal.pone.0007370 (2009). 19816605 PMC2756586

[b25] Nakamura S. *et al.* Direct metagenomic detection of viral pathogens in nasal and fecal specimens using an unbiased high-throughput sequencing approach. PLoS One 4, e4219, 10.1371/journal.pone.0004219 (2009). 19156205 PMC2625441

[b26] Breitbart M. *et al.* Metagenomic analyses of an uncultured viral community from human feces. J Bacteriol 185, 6220–6223 (2003). 14526037 10.1128/JB.185.20.6220-6223.2003 PMC225035

[b27] Frias-Lopez J. *et al.* Microbial community gene expression in ocean surface waters. Proc Natl Acad Sci USA 105, 3805–3810, 10.1073/pnas.0708897105 (2008). 18316740 PMC2268829

[b28] Breitbart M. *et al.* Genomic analysis of uncultured marine viral communities. Proc Natl Acad Sci USA 99, 14250–14255, 10.1073/pnas.202488399 (2002). 12384570 PMC137870

[b29] Shan T. *et al.* Picornavirus salivirus/klassevirus in children with diarrhea, China. Emerg Infect Dis 16, 1303–1305, 10.3201/eid1608.100087 (2010). 20678331 PMC3298310

[b30] Okamoto H. *et al.* Genomic and evolutionary characterization of TT virus (TTV) in tupaias and comparison with species-specific TTVs in humans and non-human primates. J Gen Virol 82, 2041–2050, 10.1099/0022-1317-82-9-2041 (2001). 11514713

[b31] Spandole S., Cimponeriu D., Berca L. M. & Mihaescu G. Human anelloviruses: an update of molecular, epidemiological and clinical aspects. Arch Virol 160, 893–908, 10.1007/s00705-015-2363-9 (2015). 25680568

[b32] Page R. C. & Beck J. D. Risk assessment for periodontal diseases. Int Dent J 47, 61–87 (1997). 9448791 10.1111/j.1875-595x.1997.tb00680.x

[b33] Bilder L., Elimelech R., Szwarcwort-Cohen M., Kra-Oz Z. & Machtei E. E. The prevalence of human herpes viruses in the saliva of chronic periodontitis patients compared to oral health providers and healthy controls. Arch Virol 158, 1221–1226, 10.1007/s00705-013-1609-7 (2013). 23381395

[b34] Thomasini R. L., Bonon S. H., Durante P. & Costa S. C. Correlation of cytomegalovirus and human herpesvirus 7 with CD3+ and CD3+ CD4+ cells in chronic periodontitis patients. J Periodontal Res 47, 114–120, 10.1111/j.1600-0765.2011.01413.x (2012). 21895663

[b35] Slots J. Oral viral infections of adults. Periodontol 2000 49, 60–86, 10.1111/j.1600-0757.2008.00279.x (2009). 19152526

[b36] Ninomiya M. *et al.* Analysis of the entire genomes of torque teno midi virus variants in chimpanzees: infrequent cross-species infection between humans and chimpanzees. J Gen Virol 90, 347–358, 10.1099/vir.0.007385-0 (2009). 19141443

[b37] Hino S. & Miyata H. Torque teno virus (TTV): current status. Rev Med Virol 17, 45–57, 10.1002/rmv.524 (2007). 17146841

[b38] Lan D. *et al.* Sequence analysis of a Torque teno canis virus isolated in China. Virus Res 160, 98–101, 10.1016/j.virusres.2011.05.017 (2011). 21645561

[b39] Walton A. H. *et al.* Reactivation of multiple viruses in patients with sepsis. PLoS One 9, e98819, 10.1371journal.pone.0098819 (2014). 24919177 10.1371/journal.pone.0098819 PMC4053360

[b40] Galmes J. *et al.* Potential implication of new torque teno mini viruses in parapneumonic empyema in children. Eur Respir J 42, 470–479, 10.1183/09031936.00107212 (2013). 23060626 PMC3729974

[b41] Biagini P. *et al.* Circular genomes related to anelloviruses identified in human and animal samples by using a combined rolling-circle amplification/sequence-independent single primer amplification approach. J Gen Virol 88, 2696–2701, 10.1099/vir.0.83071-0 (2007). 17872521

[b42] Luo K. *et al.* Novel variants related to TT virus distributed widely in China. J Med Virol 67, 118–126 (2002). 11920826 10.1002/jmv.2200

[b43] Grenier G., Gagnon G. & Grenier D. Detection of herpetic viruses in gingival crevicular fluid of patients suffering from periodontal diseases: prevalence and effect of treatment. Oral Microbiol Immunol 24, 506–509, 10.1111/j.1399-302X.2009.00542.x (2009). 19832804

[b44] Flemmig T. F. & Beikler T. Control of oral biofilms. Periodontol 2000 55, 9–15, 10.1111/j.1600-0757.2010.00383.x (2011). 21134225

[b45] de Oliveira J. C. *et al.* Detection of TTV in peripheral blood cells from patients with altered ALT and AST levels. New Microbiol 31, 195–201 (2008). 18623984

[b46] Vasilyev E. V. *et al.* Torque Teno Virus (TTV) distribution in healthy Russian population. Virol J 6, 134, 10.1186/1743-422X-6-134 (2009). 19735552 PMC2745379

[b47] Ninomiya M., Takahashi M., Nishizawa T., Shimosegawa T. & Okamoto H. Development of PCR assays with nested primers specific for differential detection of three human anelloviruses and early acquisition of dual or triple infection during infancy. J Clin Microbiol 46, 507–514, 10.1128/JCM.01703-07 (2008). 18094127 PMC2238095

[b48] Hussain T., Manzoor S., Waheed Y., Tariq H. & Hanif K. Phylogenetic analysis of Torque Teno Virus genome from Pakistani isolate and incidence of co-infection among HBV/HCV infected patients. Virol J 9, 320, 10.1186/1743-422X-9-320 (2012). 23270330 PMC3573928

[b49] Botero J. E., Vidal C., Contreras A. & Parra B. Comparison of nested polymerase chain reaction (PCR), real-time PCR and viral culture for the detection of cytomegalovirus in subgingival samples. Oral Microbiol Immunol 23, 239–244, 10.1111/j.1399-302X.2007.00418.x (2008). 18402611

[b50] Allander T., Emerson S. U., Engle R. E., Purcell R. H. & Bukh J. A virus discovery method incorporating DNase treatment and its application to the identification of two bovine parvovirus species. Proc Natl Acad Sci USA 98, 11609–11614, 10.1073/pnas.211424698 (2001). 11562506 PMC58777

[b51] Phan T. G. *et al.* The fecal viral flora of wild rodents. PLoS Pathog 7, e1002218, 10.1371/journal.ppat.1002218 (2011). 21909269 PMC3164639

