# Supplementary material for: A novel species of torque teno mini virus (TTMV) in gingival tissue from chronic periodontitis patients
Source: Sci Rep. 2016 May 25;6:26739. doi: 10.1038/srep26739 (PMC4879676; doi:10.1038/srep26739)
Supplement: Supplementary Information [file srep26739-s1.pdf]

A novel species of torque teno mini virus (TTMV) in gingival tissue from chronic periodontitis patients

Yu Zhang, Fei Li, Tong-Ling Shan, Xutao Deng, Eric Delwart ,Xi-Ping Feng

Table S1. % nucleotide similarity between ORF1 of different TTMV species for ORF1

	1	2	3	4	5	6	7	8	9	10
1.TTMV-222										
2.TTMV1	60.4									
3.TTMV2	57.7	54.9								
4.TTMV3	57.5	54.9	58.9							
5.TTMV4	51.7	50.9	51.2	52.2						
6.TTMV5	50.4	49.7	46.8	48.7	57.5					
7.TTMV6	51.7	50.9	50.7	50.3	53.7	49.4				
8.TTMV7	52.0	50.3	50.9	51.5	53.7	49.1	58.8			
9.TTMV8	51.7	51.1	50.6	50.7	53.3	49.4	58.8	58.5		
10.TTMV9	49.1	51.0	50.3	50.3	51.0	49.0	56.4	56.1	62.9	

Supplementary Table S2. Comparison of periodontitis and control groups.

Group	Periodontitis group (mean±SD)	Control group (mean±SD)	*P*
Age	45.49±11.81	39.72±11.19	0.674
PD	9.25±2.08	2.15±0.97	<0.001
CAL	4.72±0.97	0.14±0.18	<0.001
GI	2.32±0.43	0.12±0.17	<0.001
PLI	2.58±0.30	0.27±0.14	<0.001

Obtained by Student's *t*-test (two groups) .[a]$P<0.05$.

SD: standard deviation.PD: pocket depth.CAL: clinical attachment loss.GI: gingival index. PLI: plaque index.

Supplementary Table S3. Comparison of periodontitis and control groups.

Group	Periodontitis group	Control group	*P*
	N	N	
Gender	150	150	
Male	70	74	0.729
Female	80	76	
Frequency of BOP	150	150	
Positive	150	53	<0.001
Negative	0	97	

Obtained by Chi-squared test. [a]$P < 0.05$

BOP: bleeding on probing.
